# Morphological and biochemical responses of *Macrotyloma uniflorum* (Lam.) Verdc. to allelopathic effects of *Mikania micrantha* Kunth extracts

**DOI:** 10.1016/j.heliyon.2021.e07822

**Published:** 2021-08-17

**Authors:** Pallavi Jali, Ipsita Priyadarsini Samal, Sameer Jena, Gyanranjan Mahalik

**Affiliations:** a Department of Botany, Utkal University, Bhubaneswar, India; b Department of Botany, School of Applied Sciences, Centurion University of Technology and Management, Odisha, India

**Keywords:** Allelopathy, *Mikania micrantha*, *Macrotylama uniflorum*, Phenolics, Proline, Secondary metabolites

## Abstract

Yield loss due to noxious weeds is one among several reasons for the reduced economy for the developing countries. Impacts of one such weed i.e. *Mikania micrantha* were investigated on the rate of seed germination, growth, biomass, photosynthetic pigments, total soluble protein, phenolics and proline content of leaves of *Macrotylama uniflorum* (an important pulse). In a completely randomized setup, control and four concentrations (10 mg/ml, 50 mg/ml, 100 mg/ml and 200 mg/ml) of the aqueous leaf extracts of *M. micrantha* were tested on the seeds of *M. uniflorum*. The extracts inhibited germination, growth, biomass, chlorophyll, carotenoid and protein contents. The protein content of *M. uniflorum* decreased to 8.48 mg/g at 200 mg/ml. Similarly, shoot length and root length were also decreased up to 5.11 cm and 0.85 cm respectively and water content increased with the increasing concentration of weed extracts. The leaf extracts resulted in an increase in the phenolics (19.66 mg) and proline (24.49 mg) content of the crop plant. The preliminary study indicated that the aqueous leaf extracts of weed plant resulted in negative or detrimental impact on growth and physiology of the plant and this might be due to the release of secondary metabolites. The present investigation may further lead to the identification of certain secondary metabolites or allelo-chemicals that may have an important agricultural application for sustainability and may enhance the level of crop protection against several other harmful plant species.

## Highlights

• Allelopathic impacts of *Mikania micrantha* Kunth were investigated on the rate of seed germination, growth, biomass, photosynthetic pigments, total soluble protein, phenolics and proline content of *Macrotylama uniflorum* (Lam.) Verdc.
• The extracts inhibited germination, growth, biomass, chlorophyll, carotenoid and protein contents. The protein content of *M. uniflorum* decreased to 8.48 mg/g at 200 mg/ml. Similarly shoot length, root length also decreased up to 5.11 cm, 0.85 cm respectively and water content increased with increasing concentration of weed extracts.
• The leaf extracts resulted in an increase in the phenolics (19.66 mg) and proline (24.49 mg) content of the crop plant. The aqueous extracts of leaves caused detrimental impact on growth and physiology of the crop plant and this might be due to release of secondary metabolites.
• This present investigation may further lead to identification of certain secondary metabolites or allelo-chemicals for agricultural application and might enhance the level of crop protection against several other harmful plant species.

## 1 Introduction

A biological phenomenon by virtue of which an organism produces biochemical substances which influence the morphology and physiology of another organism is referred to as allelopathy (Cheng and Cheng, 2015). Root exudation, leaching, deposition of leaf particles and volatilization are the process involved in transport of the chemicals from one plant to another (Liza and Ram, 2017). The biochemicals or allelo-chemicals are secondary metabolites which are not required for growth, development and reproduction (Stamp, 2003; Langenheim, 1994). They could have detrimental effects on the target organisms. Allelo-chemicals are an important part of defense mechanisms in plants such as herbivory, microbial attack etc. (Stamp, 2003; Fraenkel, 1981).

*Macrotyloma uniflorum* belongs to family Fabaceae or leguminosae. It is a short day, twining, succulent and annual climbing herb. *M. uniflorum* has excellent nutritional as well as ethnomedicinal properties (Kumar, 2006; Singh, 1991; Ranasinghe and ERHSS, 2017). It has various properties like anti-obesity, anti-diabetic, antioxidant etc. (Kaundal et al., 2019). It is a rich source of vitamins, proteins and minerals used in developing countries to meet nutritional requirements.

*Mikania micrantha* is an invasive species which is responsible to alter the morphology and physiology of indigenous crop plants (Stamp, 2003; Matawali et al., 2016a, Matawali et al., 2016b). *Mikania micrantha* (weed plant) is known to grow in such a way that other plant growth and activities are retarded. It is known to release substances that hamper the growth and development of other plant species (Tiwari et al., 2005). In this way, the weed plants deteriorate young plantations as well as nurseries which have become a threat to today's sustainable agriculture system. In the current study, for exploring the allelopathic potential of *M. micrantha*, aqueous leaf extracts were used against an important ethnomedicinal pulse *Macrotyloma uniflorum*. The biochemical changes of the crop plant (*Macrotyloma uniflorum*) were recorded to verify the negative impact of *Mikania micrantha*. Allelopathic impacts may be studied further as an important mechanism to know the chemicals or metabolites involved and also may be utilized to inhibit some unwanted growth of plant species. This approach may further lead to sustainable agriculture and crop protection.

### 1.1 Study of different weeds on plant system

**Table:** 

Allelopathic plant	Effect on different plant systems
Black walnut	reduced corn yield
Leucaena	reduced the yield of wheat and turmeric
Lantana	germination and growth of milkweed vine
Sour orange	inhibited seed germination and root growth of pigweed, bermudagrass, and lambsquarters
Red maple, swamp chestnut oak, sweet bay, and red cedar	inhibited lettuce seed
Chaste tree or box elder	retarded the growth of pangolagrass, a pasture grass

## 2 Materials and methods

The experiments were conducted to study the potential allelopathic effects of *M. micrantha* on *M. uniflorum*.

### 2.1 Collection of plant materials

**Figure 1:**
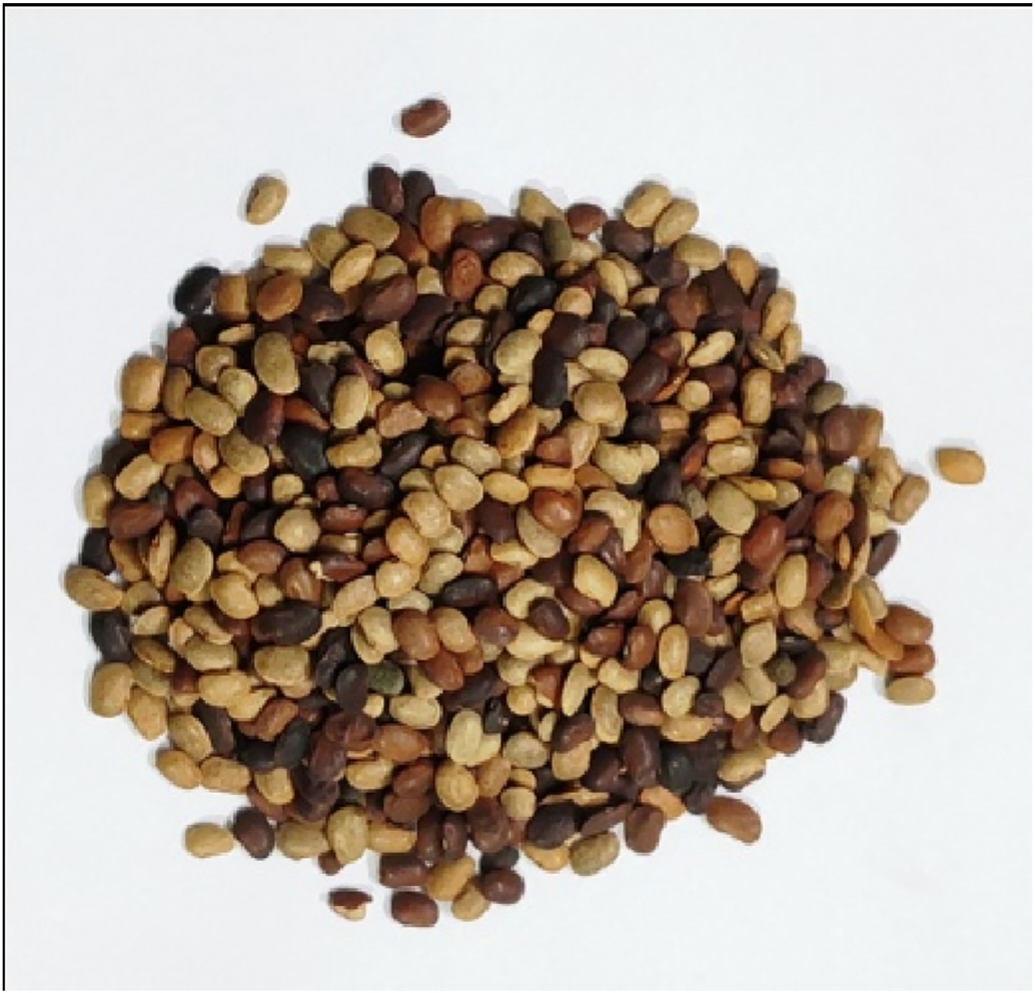
Seeds of *Macrotylama uniflorum*.

The weed *Mikania micrantha* were collected from Centurion University of Technology and Management (CUTM), Jatni campus, Odisha, India (Sahoo and Mahalik, 2020). The high quality and viable seeds of *Macrotyloma uniflorum* were collected from the Plant Breeding and Genetics department, Orissa University of Agriculture and Technology (OUAT), Odisha (Figure 1).

### 2.2 Preparation of aqueous extracts of plant material

Plant leaves were separated from the shoots and were shade dried until removal of moisture. Samples were crushed to powdered form and stored in the airtight glass jars until further use. Sample solutions of varied concentrations with increasing order (10, 50, 100 and 200 mg/ml) were prepared and stored at 4^ᵒ^C in a refrigerator and used within 24–48 h to treat the *M. uniflorum* seeds.

### 2.3 Seed germination assay and screening

Healthy and fresh seeds of *M. uniflorum* were kept on moist filter paper soaked with different concentrations of *M. micrantha* aqueous extracts. Controls were treated with 20 ml of distilled water (Bhatt et al., 2016; Mittra et al., 2004). The seeds were kept under dark for four days in a controlled room temperature. The germination was considered when the radicals were ~ 2 mm or more in length. The radical length was measured after 4 days of germination. The seedling vigour index (Abdul-Baki and Anderson, 1973), plant tolerance index (Turner and Marshall, 1972) and phytotoxicity percent (Chou and Lin, 1976) were calculated as follows:

SVI = Germination percentage x Radical length

PTI = Radical length of the treatment/ radical length of control x 100

Phytotoxicity percent = Root length of control-root length of treatment x 100

### 2.4 Water content and biomass

Biomass was determined after 14 days of treatment with aqueous extracts of *Mikania micrantha* by taking the single whole plant (*Macrotyloma uniflorum*) including shoots and roots. The seedlings were taken and properly washed with distilled water, dried using blotting paper and later the fresh weights were recorded. The samples were dried at 60 °C for 2–3 days in an oven and later the dry weights (DW) were measured (Kim et al., 2005).

### 2.5 Photosynthetic pigments

500 mg fresh and healthy leaves were properly homogenized in 80% chilled acetone. The homogenized samples were then centrifuged for 10 min at 4^ᵒ^C, 10,000 rpm in the dark. Supernatant was taken and the absorbances were recorded (Arnon, 1949).

### 2.6 Total leaf protein

The soluble proteins were extracted using Acetone-TCA method (Parida et al., 2002; Lowry et al., 1951). After the extraction, samples were centrifuged at 14,000 rpm for 20 min and the supernatant was collected. Sample (0.2 ml) was taken to which 1 ml de-ionized water was added. Further alkaline Copper (5 ml) solution was added and kept for 10 min. Folin-Ciocalteau reagent (0.5 ml) was added and then incubated for 30 min in the dark. Later the absorbance at 660 nm was recorded.

### 2.7 Total phenolics

Sample extraction was carried out using 80% ethanol and estimated using Folin – Ciocalteau reagent (Parida et al., 2002; Mallik and Singh, 1980). 0.5 g leaf samples were homogenized with 80% ethanol. The homogenates were centrifuged for 20 min at 10,000 rpm. Residues were re-extracted several times with 80% ethanol by centrifugation. The supernatant collected was evaporated and the residues after evaporation were dissolved with distilled water (5 ml). 2 ml sample was taken and the volume was made up to 3ml, by distilled water. 0.5 ml Folin-Ciocalteau reagent and 20% Sodium Carbonate (2 ml) solution was further added. The reagents were mixed properly and kept in boiling water bath for 1 min. The mixture was then cooled to room temperature and absorbance at 650 nm was recorded.

### 2.8 Proline

0.5g fresh leaves were taken and properly homogenized using 3% sulfosalicylic acid. The homogenates were filtered through filter paper. 2 ml each of the filtrate, ninhydrin and glacial acetic acid was mixed altogether and incubated at 100 °C for 1 h (Bates et al., 1973). The reaction was later stopped by keeping test tubes in an ice bucket. Toluene (2 ml) was added and the mixture was vigorously shaken for a few seconds. The separated aqueous layer of toluene was warmed at room temperature; the colored sample was measured at 520nm wavelength.

### 2.9 Statistical analysis

Data represented through mean along with the standard deviation calculated from five number of replicates and three experiments consecutively. DMRT was used as a post hoc test after running ANOVA to analyze and compare the allelopathic effect of *M. micrantha* on *M. uniflorum* at p < 0.05 (5% significance level).

## 3 Results and discussion

### 3.1 Seed germination assay and plant growth

The rate of germination of *M. uniflorum* seeds responded differently manner to different concentrations of *M. micrantha* aqueous extracts. Germination rate decreased with increasing concentration of *M. micrantha* extracts and the detrimental effect was more pronounced at 200 mg/ml concentration (Table 1). The germination percentage was observed to be 85.55% (control), 83.75% (10 mg), 76.25% (50 mg), 50.78% (100 mg) and 30.44% (200 mg). The radical length decreased with increasing aqueous extract concentrations of *M. micrantha* that accounted for 4.12 cm and 1.26 cm for 10 mg and 200 mg respectively (Table 1). The seedling vigor index of *M. uniflorum* was found to be 343.37 and 38.55 in 10 mg and 200 mg treatment respectively. Plant tolerance index was observed to show a decreasing trend from 10 mg to 200 mg aqueous extracts of *M. micrantha*. Tolerance to *M. micrantha* decreased when treatment reached to 200 mg amounting to 29.68.

**Table 1:** Germination percentage, Radical length, Seedling vigour index (SVI), Plant tolerance index (PTI) and Phytotoxicity percentage of *M. uniflorum*.

Sample	*M. micrantha* extracts (mg/ml)	Germination (%)	Radical length (cm)	SVI	PTI	% Phytotoxicity
*M. uniflorum*	0	85.55 ± 0.74^a^	4.26 ± 0.65^a^	373.54	100	0
	10	83.75 ± 0.94^a^	4.12 ± 0.61^a^	343.37	96.09	166
	50	76.25 ± 1.69^b^	2.61 ± 0.33^b^	198.25	60.93	258
	100	50.78 ± 1.69^c^	1.76 ± 0.12^c^	89.71	41.41	283
	200	30.44 ± 0.94^d^	1.26 ± 0.21^c^	38.55	29.68	329

∗Values represent mean ± SD, letters represent significant differences among treatments at 5% level of significance (P ≤ 0.05) as per the DMRT analysis.

### 3.2 Effect of *M. micrantha* on growth of *M. uniflorum*

**Figure 2:**
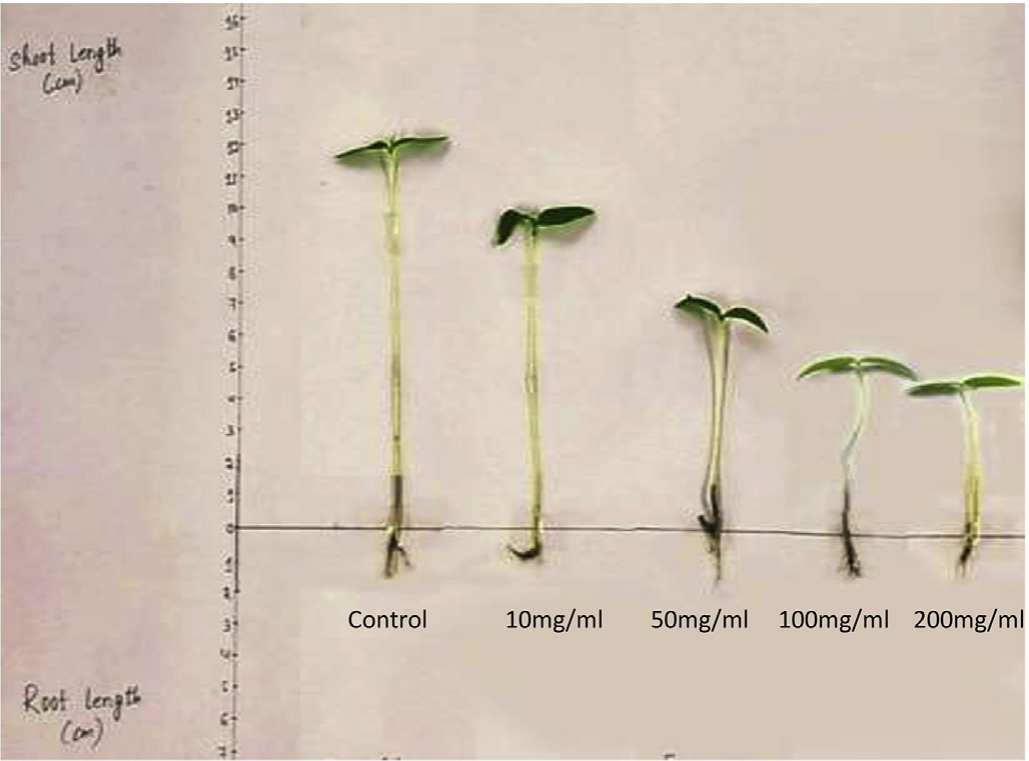
Shoot and Root length of *M. uniflorum* exposed to different concentrations of *M. micrantha* plant extracts.

*M. micrantha* extracts in the experimental conditions a showed significant amount of growth reduction in *M. uniflorum*. Growth of shoots were affected significantly at 200 mg (5.11 cm), 100 mg (5.67 cm), 50 mg (6.43 cm) and 10 mg (9.59 cm) as compared to control (12.61 cm) (Table 2, Figure 2). Root length indicated growth reduction in all the samples i.e. 2.48 cm, 1.56 cm, 1.31 cm and 0.85 cm at 10 mg, 50 mg, 100 mg and 200 mg respectively (Table 2).

**Table 2:** Effect of *M. micrantha* on growth of *M. uniflorum*.

Treatments (mg/ml)	Shoot length (cm)	Root length (cm)
Control	12.61 ± 0.16^a^	4.14 ± 0.24^a^
10	9.59 ± 0.12^b^	2.48 ± 0.19^b^
50	6.43 ± 0.19^c^	1.56 ± 0.01^c^
100 200	5.67 ± 0.21^d^ 5.11 ± 0.13^d^	1.31 ± 0.21^c^ 0.85 ± 0.07^d^

∗Values represent mean ± SD, letters represent significant differences among treatments at 5% level of significance (P ≤ 0.05) as per the DMRT analysis.

**Figure 3:**
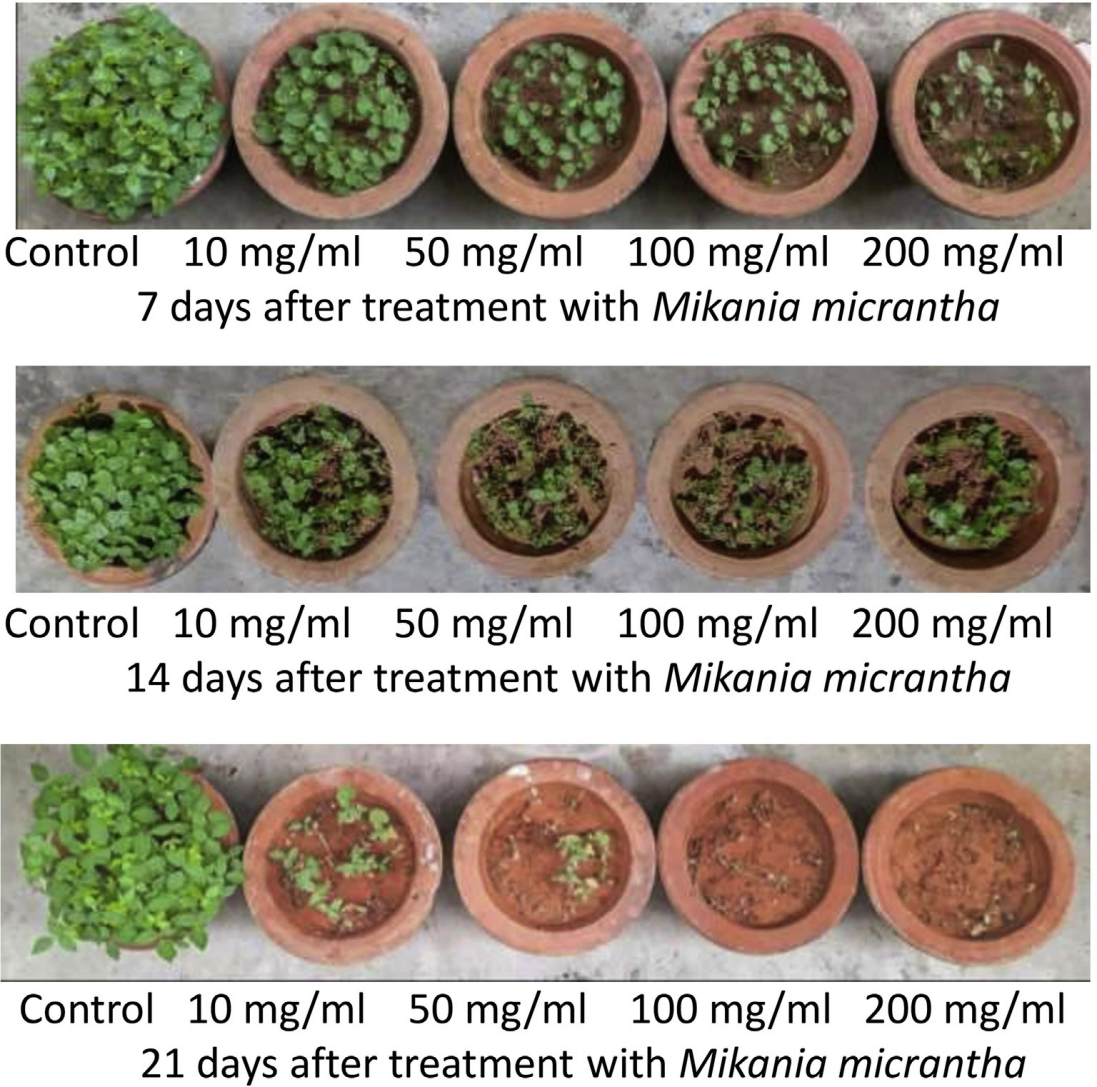
Morphological changes of *M. uniflorum* upon exposure to *M. micrantha* extracts.

Abiotic stress is known to be highly toxic and have severe deleterious impacts on the growth of the plant (Rubio et al., 1994; Watanabe and Suzuki, 2002; Maksymiec and Krupa, 2006). In the growth medium it is observed to be having significant shoot and root length reduction (Dong et al., 2005) (Figure 3). The most noticeable symptoms of abiotic toxicity were found to be the stunted growth (Huang et al., 2000; Li and Jin, 2010; Kaur and Malhotra, 2012). Similar results regarding the effects of leaf extracts of different weeds (*Parthenium hysterophorus*, *Tridax Procumbens* and *Hyptis Saveolens*) on *Vigna mungo* germination and growth were observed (Babu et al., 2014).

### 3.3 Effect of *M. micrantha* on biomass of *M. uniflorum*

*M. micrantha* extracts showed significant biomass reduction in *M. uniflorum* plants. Biomass was significantly affected by *M. micrantha* treated aqueous extracts to *Macrotyloma uniflorum* plants i.e. 10 mg (90.56%), 50 mg (89.18%), 100 mg (90.32%) and 200 mg (94.73%) as compared to control (44.25%) plants (Table 3). Growth reduction affects biomass of the plant (Ismail and Mah., 1993; Day et al., 2016). With an increase in concentrations of the weed extracts, there was a noticeable reduction of biomass of *M. uniflorum*. The water content increased with increasing concentrations of aqueous extracts.

**Table 3:** Effect of *M. micrantha* on biomass of *M. uniflorum*.

Treatments (mg/ml)	Fresh Weight (g)	Dry Weight (g)	Water Content (%)
Control	0.62 ± 0.26^a^	0.11 ± 0.14^a^	82.25
10	0.53 ± 0.22^b^	0.05 ± 0.11^b^	90.56
50	0.37 ± 0.09^c^	0.04 ± 0.11^b^	89.18
100 200	0.31 ± 0.11^c^ 0.19 ± 0.03^d^	0.03 ± 0.31^b^ 0.01 ± 0.27^c^	90.32 94.73

∗Values represents mean ± SD, letters represent significant differences among treatments at 5% level of significance (P ≤ 0.05) as per the DMRT analysis.

### 3.4 Changes in concentrations of photosynthetic pigments

**Figure 4:**
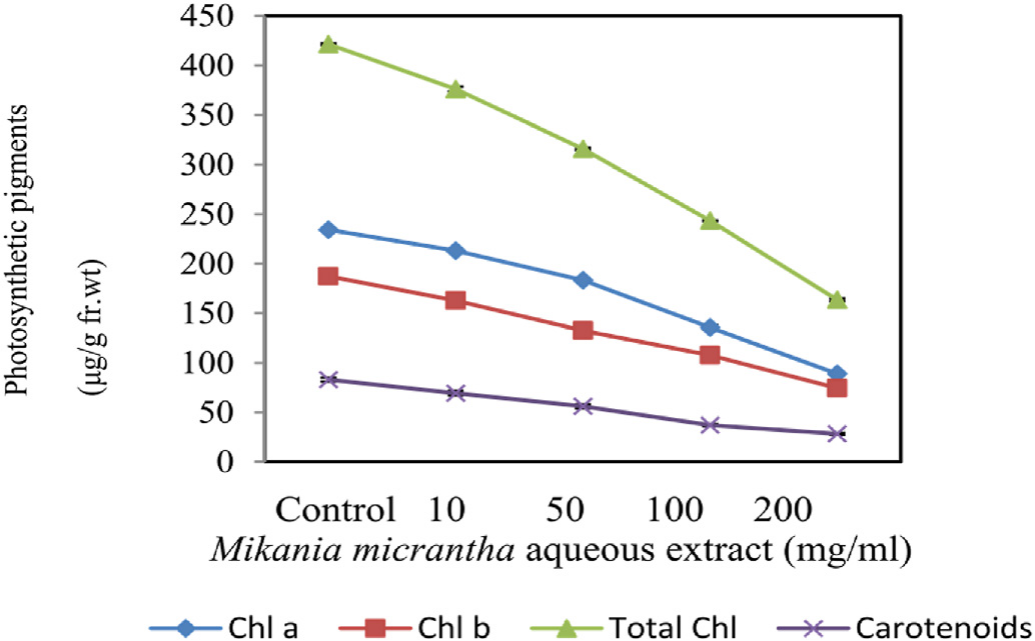
Photosynthetic pigments of *M. uniflorum* exposed to varying concentrations of *M. micrantha* extracts.

The quantity of pigments (Chl *a*, Chl *b*, total chlorophyll and carotenoids) under different *M. micrantha* aqueous extract concentrations were studied. Total Chlorophyll and carotenoid contents of *M. uniflorum* decreased significantly with an increase in *M. micrantha* levels compared to control plants (Figure 4). The Chl *a*, Chl *b* and carotenoid contents showed decreasing effects upon exposure to *M. micrantha* extracts. Chl *a* contents were recorded 234.31 μg/g and 89.07 μg/g at 10 mg/ml and 200 mg/ml respectively. Carotenoids showed 28.19 μg/g, 37.02 μg/g, 56.11 μg/g and 69.42 μg/g upon exposure to 200, 100, 50, 10 mg/ml of extracts of *M. micrantha* respectively whereas control carotenoid levels showed 83.13 μg/g.

Extracts of *M. micrantha* effectively inhibit the photosynthetic pigments of *M. uniflorum* at various concentrations. This might be due to the release of secondary metabolites (CEN et al., 2004; Huang et al., 2012; Jyothilakshmi et al., 2015; Matawali et al., 2016a, Matawali et al., 2016b) that resulted in the reduction or inhibition of the photosynthetic activity or destruction of some chloroplasts. It was observed that Chl *a*, Chl *b* and carotenoid contents significantly decreased due to reduction in cellular Mg^2+^ ion concentration, which is essential for the biosynthesis of chlorophyll (Yildirim et al., 2008; Mohamed and Gomaa, 2012). Earlier it was reported that a decrease in the chlorophyll content might be due to failure in chlorophyll biosynthesis or disruption of some chloroplasts (Padmaja et al., 1990; Ahmad et al., 2007). The similar decreasing trend in the photosynthetic activity was also observed upon treatment of *Amaranthus viridis* extracts on *Triticum aestivum* (Shinde and Salve, 2019; Patel and Kumbhar, 2016).

### 3.5 Changes in soluble protein content

**Figure 5:**
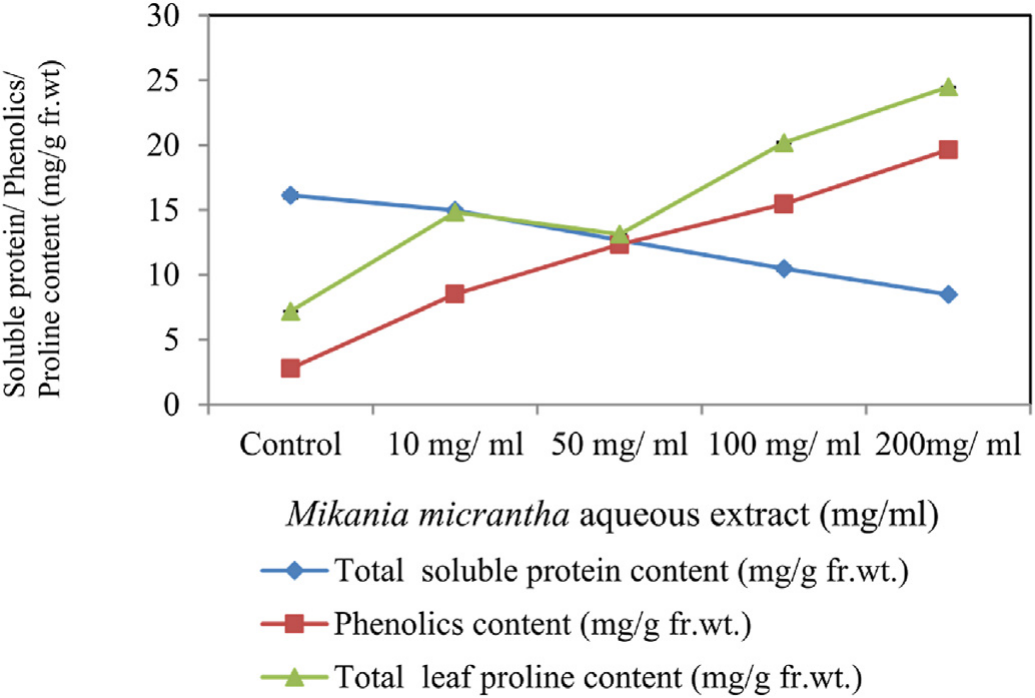
Total soluble protein content, Phenolics and Proline content of *M. uniflorum* exposed to different concentrations of *M. micrantha* aqueous extracts.

Total soluble leaf protein of *M. uniflorum* (obtained in mg/ml fr wt.) showed remarkable variations when treated with different extracts of this weed (Figure 5) 14.96 mg/g, 12.69 mg/g, 10.48 mg/g and 8.48 mg/g at 10, 50, 100 and 200 mg/ml respectively as compared to control plants (16.63 mg/g). The above results showed that upon exposure to stress, plants show reduced protein levels as compared to the control.

The total protein contents reduced significantly as the result of exposure to *M. micrantha* plant extracts in *M. uniflorum*. The decline in protein level may be due to the disruption in translation pathway after being exposed to the allelochemicals released. This phenomenon was also observed in plants exposed to both biotic and abiotic stress (Parida et al., 2004; Jali et al., 2019).

### 3.6 Changes in phenolics and proline contents

Control plants showed minute phenolic content i.e. 2.79 mg/g. The phenolic contents in treated samples of *M. uniflorum* increased up to 8.51 mg/g, 12.35 mg/g, 15.46 mg/g and 19.66 mg/g at 10, 50, 100 and 200 mg/ml aqueous extracts respectively which is significantly higher than control (Figure 5). Enhanced phenolic metabolism produces antioxidative activity which focuses to decrease the toxic and negative effects of the stress (Zheng et al., 2011; Dai et al., 2006; Kovacsluik and Backor, 2007). Extracts of *M. micrantha* slow down the germination and growth of a number of plant species (Zhang et al., 2002). At least three sesquiterpenoids (secondary metabolites) have been recognized that produce this effect (Shao et al., 2005; Matawali et al., 2016). Low dose of phenolic compounds stimulates protein synthesis and activation of antioxidant enzymes (Baziramakenga et al., 1995) which are effective in plant protection (Kleiner et al., 1999), while high levels of phenolic application result in plant damage (Politycka et al., 2004). Proline accumulation in leaf tissues wase more pronounced with an increase in *M. micrantha* treated samples of *M. uniflorum*. In *M. uniflorum*, the maximum proline level was reported at 200 mg/ml (24.49 mg/g) followed by 100 mg (20.19 mg/g), 50 mg/ml (13.13 mg/g) and 50 mg/ml (14.83 mg/g) (Figure 5). Proline accumulation is a general phenomenon in all stressed plants (Saradhi and Vani, 1993; Lee and Liu, 1999; Hernandez et al., 2000; Dhir et al., 2004; Ahmad et al., 2006; Koca et al., 2007; Parida and Jha, 2010; Shahbaz et al., 2013). Proline also acts as a major reservoir of nitrogen and energy, that can be utilised in resuming the growth of the plant after removal of the stress (Chandrashekar and Sandhyarani, 1996).

## 4 Conclusions

The rate of seed germination, plant growth, biochemical parameters of *M. uniflorum* increased in control conditions as compared with plant exposed to different concentrations of *M. micrantha* extract. There was a noticeable reduction in seed germination, plant growth (shoot and root length), photosynthetic pigments and protein content in plants treated with weed extracts, while there was an increase in total phenolics and proline content. The decrease of biomolecules of *M. uniflorum* in the present study might be due to the release of secondary metabolites from *M. micrantha*. The increase in phenolics and proline content might be due to the inherent capacity of the plants to respond to different stress conditions. Higher the amount of phenolics and proline, higher the level of stress builds up in the plant system. In the present study, the weed extracts inhibit the crop plant's growth and physiology. This study will definitely lead to the identification of few phytocompounds or metabolites having beneficial agricultural applications for sustainability in near future. Sensitivity to the allelo-chemicals and extent of inhibition vary from crop to crop but it will enhance future aspects of crop protection from several harmful plant species.

## Declarations

### Author contribution statement

Pallavi Jali; Gyanranjan Mahalik: Conceived and designed the experiments; Analyzed and interpreted the data; Contributed reagents, materials, analysis tools or data; Wrote the paper.

Ipsita Priyadarsini Samal; Sameer Jena: Performed the experiments; Wrote the paper.

### Funding statement

This research did not receive any specific grant from funding agencies in the public, commercial, or not-for-profit sectors.

### Data availability statement

The authors do not have permission to share data.

### Declaration of interests statement

The authors declare no conflict of interest.

### Additional information

No additional information is available for this paper.
